# Neuroprotective Effects of Growth Hormone (GH) and Insulin-Like Growth Factor Type 1 (IGF-1) after Hypoxic-Ischemic Injury in Chicken Cerebellar Cell Cultures

**DOI:** 10.3390/ijms22010256

**Published:** 2020-12-29

**Authors:** Rosario Baltazar-Lara, José Ávila-Mendoza, Carlos G. Martínez-Moreno, Martha Carranza, Santiago Pech-Pool, Olivia Vázquez-Martínez, Mauricio Díaz-Muñoz, Maricela Luna, Carlos Arámburo

**Affiliations:** 1 Departamento de Neurobiología Celular y Molecular, Instituto de Neurobiología, Campus Juriquilla, Universidad Nacional Autónoma de México, Querétaro 76230, Mexico; mabal92@comunidad.unam.mx (R.B.-L.); javila@comunidad.unam.mx (J.Á.-M.); cgmartin@comunidad.unam.mx (C.G.M.-M.); macasa@unam.mx (M.C.); agronomiapech@hotmail.com (S.P.-P.); ovazquez@comunidad.unam.mx (O.V.-M.); mdiaz@comunidad.unam.mx (M.D.-M.); 2 Department of Molecular, Cellular and Developmental Biology, The University of Michigan, Ann Arbor, MI 48109, USA

**Keywords:** hypoxia-ischemia, growth hormone, IGF-1, cerebellum, neuroprotection

## Abstract

It has been reported that growth hormone (GH) and insulin-like growth factor 1 (IGF-1) exert protective and regenerative actions in response to neural damage. It is also known that these peptides are expressed locally in nervous tissues. When the central nervous system (CNS) is exposed to hypoxia-ischemia (HI), both GH and IGF-1 are upregulated in several brain areas. In this study, we explored the neuroprotective effects of GH and IGF-1 administration as well as the involvement of these endogenously expressed hormones in embryonic chicken cerebellar cell cultures exposed to an acute HI injury. To induce neural damage, primary cultures were first incubated under hypoxic-ischemic (<5% O_2_, 1g/L glucose) conditions for 12 h (HI), and then incubated under normal oxygenation and glucose conditions (HI + Ox) for another 24 h. GH and IGF-1 were added either during or after HI, and their effect upon cell viability, apoptosis, or necrosis was evaluated. In comparison with normal controls (Nx, 100%), a significant decrease of cell viability (54.1 ± 2.1%) and substantial increases in caspase-3 activity (178.6 ± 8.7%) and LDH release (538.7 ± 87.8%) were observed in the HI + Ox group. On the other hand, both GH and IGF-1 treatments after injury (HI + Ox) significantly increased cell viability (77.2 ± 4.3% and 72.3 ± 3.9%, respectively) and decreased both caspase-3 activity (118.2 ± 3.8% and 127.5 ± 6.6%, respectively) and LDH release (180.3 ± 21.8% and 261.6 ± 33.9%, respectively). Incubation under HI + Ox conditions provoked an important increase in the local expression of GH (3.2-fold) and IGF-1 (2.5-fold) mRNAs. However, GH gene silencing with a specific small-interfering RNAs (siRNAs) decreased both GH and IGF-1 mRNA expression (1.7-fold and 0.9-fold, respectively) in the HI + Ox group, indicating that GH regulates IGF-1 expression under these incubation conditions. In addition, GH knockdown significantly reduced cell viability (35.9 ± 2.1%) and substantially increased necrosis, as determined by LDH release (1011 ± 276.6%). In contrast, treatments with GH and IGF-1 stimulated a partial recovery of cell viability (45.2 ± 3.7% and 53.7 ± 3.2%) and significantly diminished the release of LDH (320.1 ± 25.4% and 421.7 ± 62.2%), respectively. Our results show that GH, either exogenously administered and/or locally expressed, can act as a neuroprotective factor in response to hypoxic-ischemic injury, and that this effect may be mediated, at least partially, through IGF-1 expression.

## 1. Introduction

It is clearly established that the growth hormone (GH)/insulin-like growth factor-1 (IGF-1) axis is involved in the regulation of somatic growth and several metabolic processes in vertebrates. It is also known that these growth factors participate in the control of neurogenesis, gliogenesis, myelinization, and brain plasticity, as well as in the proliferation of neural precursors [1,2,3] during the development of the central nervous system (CNS), and that they are implicated in the modulation of diverse neural and brain functions, such as cognition, learning, memory, neuroprotection, and regeneration, among others [4,5,6].

Many reports have shown that both GH and IGF-1 are also expressed in several brain areas (e.g., hypothalamus, hippocampus, cortex, and cerebellum) [7,8,9], so it is possible that the effect of these factors upon some CNS functions may be the result of a complex interaction between endocrine, paracrine, and autocrine mechanisms. Several studies have described that both circulating and locally expressed GH and IGF-1 are involved in neuroprotective actions in response to neural injury [4,10,11,12,13,14,15,16,17,18,19]. Furthermore, it was reported that GH and IGF-1 provoked an increase in the number of progenitor cells, neurons, oligodendrocytes, and astrocytes in the brain, as well as in angiogenesis, thus augmenting the cerebral blood supply [2,20]. It was also suggested that at least part of the observed neuroprotective effects can be the result of a rise in the local GH and IGF-1 expression, which occurs in damaged tissues following brain injury. Previous studies reported that exogenous GH administration increased the cell viability of cultured cerebellar neurons exposed to hypoxic conditions, and that it prevented apoptosis by triggering the PI3K/Akt pathway, inhibiting caspase-3 activity, and inducing Bcl-2 expression [11]. Likewise, the participation of GH and IGF-1 in the neuroprotective mechanisms that occur after neural harm has been described in the CNS of rats, both in vivo and in vitro [14,20]; this was also reported in chicken neuroretina, where endogenously expressed GH exerted antiapoptotic actions and promoted the survival of retinal ganglion cells (RGCs) [21,22,23]. Similarly, the neuroprotective actions of IGF-1 in response to a hypoxia-ischemia (HI) injury, both in vivo and in vitro, involve the activation of PI3K/Akt and MAP/ERK pathways, the inactivation of glycogen synthase kinase 3 beta (GSK3ß), and a concomitant reduction in caspase-3 and caspase-9 activities [24,25,26]. In addition to its protective actions, GH was also implicated in the regenerative response of neurons [20], as seen by the induction of neural outgrowths in primary neuroretinal cell cultures after a kainate-induced excitotoxic injury [17].

The upregulation of endogenous GH and IGF-1 expression observed in response to an HI neural injury has been suggested as part of the neuroprotective mechanism [27,28]. Accordingly, when either organotypic or primary cerebellar cell cultures were exposed to HI conditions, a significant increase in local GH expression was reported [11], and in vivo studies showed that a reduction of damage after brain HI injury correlated with an increase in the local expression of IGF-1 [29]. This upregulation has been causally linked to events occurring during the subacute phase of HI injury [28,30].

Whether GH neuroprotective actions are exerted directly or mediated by IGF-1 still remains controversial [31]. Moreover, the contribution of circulating versus locally expressed GH or IGF-1 and their role in neuroprotection in response to a neural harm deserve further research. To this end, in this work we study the effects on cell viability, signaling pathways, apoptosis, and necrosis when treating primary cerebellar cell cultures exposed to acute HI injury and reoxygenation (HI + Ox) with either exogenously added GH and IGF-1, or by knocking-down the endogenous expression of GH and IGF-1 genes using specific small-interfering RNAs (siRNAs), to determine their influence on the neuroprotective response to neural damage.

## 2. Results

**Figure 1:**
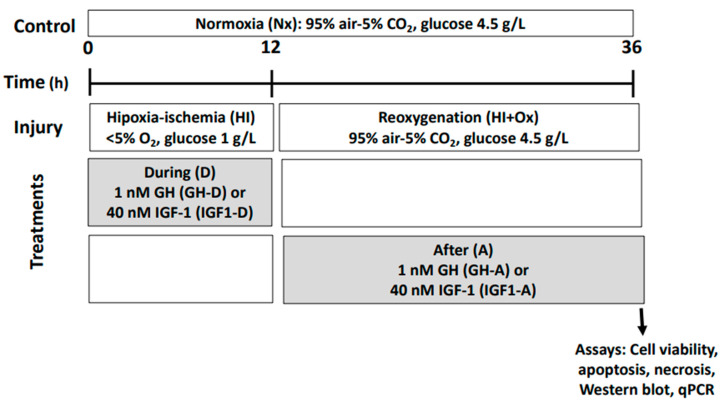
Schematic representation of the experimental design. Primary cerebellar cell cultures were first incubated under hypoxic-ischemic (HI; <5% O_2_, glucose 1 g/L) conditions for 12 h, and then incubated under normal oxygenation and glucose conditions for another 24 h (reoxygenation, HI + Ox). Growth hormone (GH) and insulin-like growth factor 1 (IGF-1) treatments were added either during HI conditions or after in HI + Ox (D denotes during, A denotes after); their effect on cell viability (MTT assay), apoptosis (Caspase-3 activity assay), necrosis (LDH activity), immunoreactivity of several markers (i.e., NeuN, DCX, pAkt, pERK1/2, Bcl-2 by Western blot), and expression of GH, GHR, IGF-1 and IGF-1R mRNAs by qPCR was evaluated.

Figure 1 depicts the experimental design and time frame employed to study the neuroprotective effects of GH and IGF-1 in cerebellar cell cultures exposed to hypoxia-ischemia (HI) for 12 h and followed by reoxygenation conditions (HI + Ox) for another 24 h.

### 2.1. Hypoxic-Ischemic Conditions Increase HIF-1α Expression in Primary Cerebellar Cell Cultures

**Figure 2:**
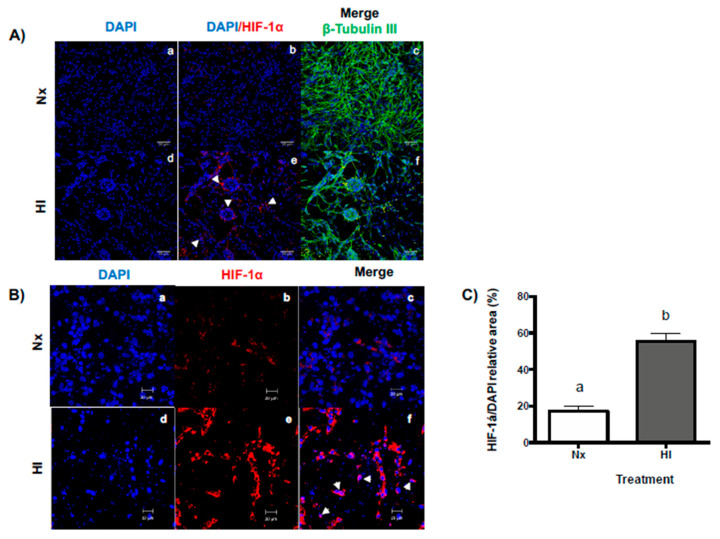
Expression of hypoxia-inducible factor-1 alpha (HIF-1α) and cell organization in primary cerebellar cultures exposed to either normoxic (Nx, 95% air-5%CO_2_, 4.5 g/L glucose) or hypoxic-ischemic (HI, <5% O_2_, 1 g/L glucose) incubation conditions for 12 h. (**A**) Representative ICC confocal images show the expression of HIF-1α (red), the neuronal marker ß-Tubulin III (green), and cell nuclei counterstained with DAPI (blue), under Nx (**Aa**–**Ac**) and HI (**Ad**–**Af**) conditions. HIF-1α immunoreactivity was enhanced after HI exposure (white arrows, **Ae**) in comparison to Nx (**Ab**). In addition, in contrast to regular cell distribution in the Nx controls, an important proportion of cell clumping and reorganization of ß-Tubulin III was observed in HI-injured cultures. Scale bars = 50 µm. (**B**) Representative ICC confocal images show the effect of HI upon the cell nuclei number (DAPI, blue) and HIF-1α (red) expression under Nx (**Ba**–**Bc**) and HI (**Bd**–**Bf**) incubation conditions. Merged images show that hypoxia-ischemia injury provoked an important reduction in cell number as well as a significant enhancement of HIF-1α immunoreactivity, mainly in the perinuclear areas (white arrows, **Bf**) in comparison to the Nx controls (**2Bc**). Scale bars = 20 µm. (**C**) Quantification of data in (**B**) is presented as the percentage (%) of the ratio between HIF-1α and DAPI fluorescence. Each bar represents the mean ± SEM, *n* = 4 independent experiments performed in duplicate. Different letters indicate significant difference (*p <* 0.0001) as determined by an unpaired Student’s *t* test.

As it is shown in Figure 2, immunocytochemical confocal analysis revealed that relevant morphological changes occurred when cerebellar cell cultures were exposed to hypoxic-ischemic conditions (HI, <5% O_2_; 1 g/L glucose) in comparison with normoxic conditions (Nx, 95% air-5% CO_2_; 4.5% g/L glucose). These changes are as follows: (1) The number of cells (estimated by DAPI staining, blue) clearly decreased (Figure 2Bd vs. Figure 2Ba); (2) a significant increase in cell clumping indicating a rise in cell lysis (white arrowheads Figure 2Ae,f vs. Figure 2Ab,c) was observed; (3) modification of ß-tubulin III organization within neural cells (Figure 2Af vs. Figure 2Ac) was evident; and (4) the immunoreactivity (IR) to hypoxia-inducible factor 1-alpha (HIF-1α) significantly increased (3-fold, *p* < 0.0001, Figure 2C) in the HI-exposed cultures (Figure 2Ae,Be,Bf). The HIF-1α-IR was abundantly present in the cytoplasm (Figure 2Be,f) and the perinuclear areas (Figure 2Bf, white arrows) of the cells exposed to HI for 12 h in comparison with Nx (Figure 2Bb,c).

### 2.2. Effects of Hypoxic-Ischemic (Acute Injury) and Reoxygenation (Subacute Injury) Incubation Conditions on Cell Viability, Apoptosis, and Necrosis in Primary Cerebellar Cultures

Supplementary Figure S1A shows that the viability of cerebellar cell cultures significantly decreased (24.1 ± 6.3%, *p* < 0.001) when exposed to HI conditions for 12 h in comparison to the Nx controls (100 ± 2.7%). When the cultures were returned to reoxygenation conditions (after HI) in a Neurobasal B-27 medium (HI + Ox) for another 24 h, the cell viability was further diminished (50.3 ± 4.2%), both in comparison to Nx (*p <* 0.0001) and to HI (*p <* 0.01) groups. Accordingly, incubation under both HI and HI + Ox conditions significantly increased the apoptosis in cerebellar cultures, as determined by caspase-3 activity (203.9 ± 12.9%, *p <* 0.0001, and 173.8 ± 13.4%, *p <* 0.001, respectively) in comparison to Nx controls (100 ± 7.1%) (Supplementary Figure S1B). Moreover, a drastic elevation in cell necrosis, as revealed by LDH release, was observed mainly under HI + Ox conditions (538.7 ± 87.8%, *p* < 0.0001) in comparison to Nx (100 ± 4.5%) and HI (152.4% ± 8.2, *p* < 0.006) (Supplementary Figure S1C).

### 2.3. Treatments with GH and IGF-1 Protect Primary Cerebellar Cell Cultures from Damage When Administered during or after HI and HI + Ox Incubation Conditions

**Figure 3:**
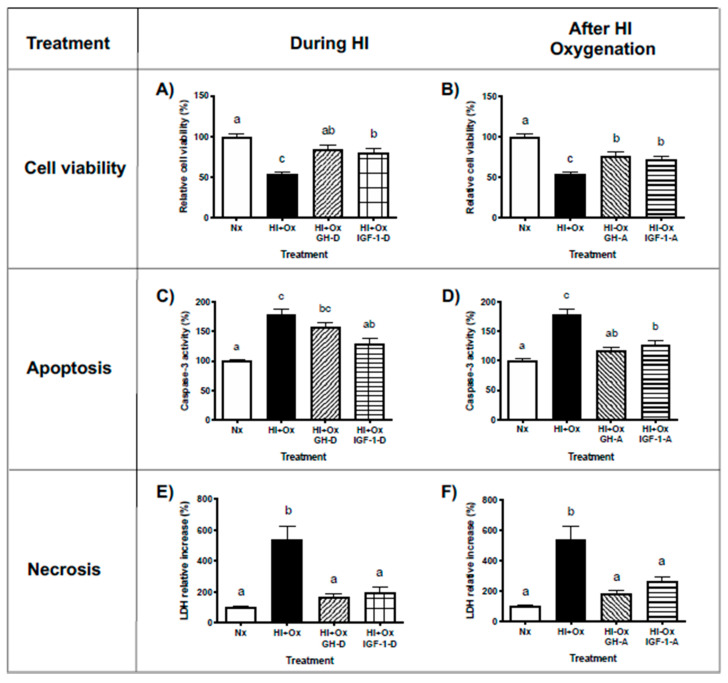
Effects of GH or IGF-1 treatments upon cell viability, apoptosis, and necrosis in primary cerebellar cultures exposed to Nx, HI, or HI + Ox incubation conditions. The hormones (1 nM GH or 40 nM IGF-1) were administered either during (denoted by D) the acute 12 h injury phase (HI, panels (**A**,**C**,**E**)) or after it (denoted by A); this occurred in the subacute phase in the reoxygenation 24 h period (HI + Ox, panels (**B**,**D**,**F**)). Panels (**A**,**B**) show the analysis of cell viability as determined by the MTT assay. Panels (**C,D**) show the evaluation of apoptosis, measuring caspase-3 activity. Panels (**E**,**F**) show cell necrosis, as determined by LDH activity released to the culture media. Values are reported as percentage change in relation with the normoxic (Nx) control groups (100%). Both hormones were capable of recovering cell viability (panels (**A**,**B**)) and decreasing cell necrosis (panels (**E**,**F**)) when administered either during the acute injury phase (HI + Ox GH-D; HI + Ox IGF-1-D) or in the reoxygenation period (HI + Ox GH-A; HI + Ox IGF-1-A). On the other hand, IGF-1 treatment substantially reduced caspase-3 activity in both conditions (panels (**C**,**D**)), whereas GH treatment was only effective when added in the subacute injury phase (HI + Ox GH-A). Bars represent the mean ± SEM, *n* = 5 independent experiments performed in triplicate. Groups with different letters are significantly different by one-way ANOVA and Tukey’s post hoc test *(p* < 0.01).

The potential effects of GH and IGF-1 to protect cells from damage were studied when these hormones were administered either during (denoted by D) the acute phase of hypoxia (HI, 12 h) or after it (denoted by A) while they are in the subacute phase of injury in the reoxygenation incubation period (HI + Ox, 24 h), as determined by analyzing their impact on cell viability (MTT assay), apoptosis (caspase-3 activity), and necrosis (LDH release) in primary cerebellar cell cultures. As it is shown in Figure 3A,B, cell viability decreased significantly under HI + Ox conditions (54.1 ± 2.1%) in comparison to Nx controls (100 ± 3.7%). Interestingly, treatment with 1 nM rcGH provoked a significant recovery of cell viability either when administered during the acute phase (HI + Ox GH-D, 84.4 ± 5.4%, *p* < 0.0001) or in the subacute phase (HI + Ox GH-A, 77.2 ± 4.2%, *p* < 0.0001) of injury. On the other hand, caspase-3 activity clearly increased under HI + Ox incubation conditions (178 ± 8.7%) compared to Nx (100 ± 2.9%), while the addition of rcGH prevented apoptosis significantly (118.2 ± 3.8%, *p* < 0.0001) only when applied in the reoxygenation period (HI + Ox GH-A, Figure 3C,D). Furthermore, necrosis was significantly augmented under HI + Ox conditions (538.7 ± 87.8%, *p* < 0.0001) in relation to Nx, whereas treatment with 1 nM rcGH appreciably blocked this effect under both GH-D (169.0 ± 19.9%, *p* < 0.01) and GH-A (180.3 ± 21.8%, *p* < 0.0002) administration protocols (Figure 3E,F, respectively).

Likewise, treatment with 40 nM rhIGF-1 promoted a significant recovery of cell viability when added either during acute (HI + Ox IGF-1-D, 79.6 ± 5.9%, *p* < 0.0004, Figure 3A) or subacute (HI + Ox IGF-1-A, 72.3 ± 3.9%, *p* < 0.01, Figure 3B) phases of injury, as compared to HI + Ox conditions alone (54.1 ± 2.1%). Moreover, the addition of IGF-1 reduced the caspase-3 activity under both conditions, i.e., HI + Ox IGF-1-D (130.2 ± 8.7%, *p* < 0.0005, Figure 3C) and HI + Ox IGF-1-A (127.5 ± 6.6%, *p* < 0.0001, Figure 3D), respectively, in comparison with HI + Ox (178.6 ± 8.7%). Furthermore, the administration of IGF-1 also elicited a significant decrease in necrosis, as determined by LDH release into the culture media, when supplemented under either HI + Ox IGF-1-D (193.6 ± 40.1%, *p* < 0.03, Figure 3E) or HI + Ox IGF-1-A (261.6 ± 33.9%, *p* < 0.01, Figure 3F), as compared with HI + Ox (538.7 ± 87.9%) incubation conditions.

### 2.4. Treatments with GH and IGF-1 Protect Mature Neurons and Neuronal Precursors after an Acute Hypoxic-Ischemic Injury

**Figure 4:**
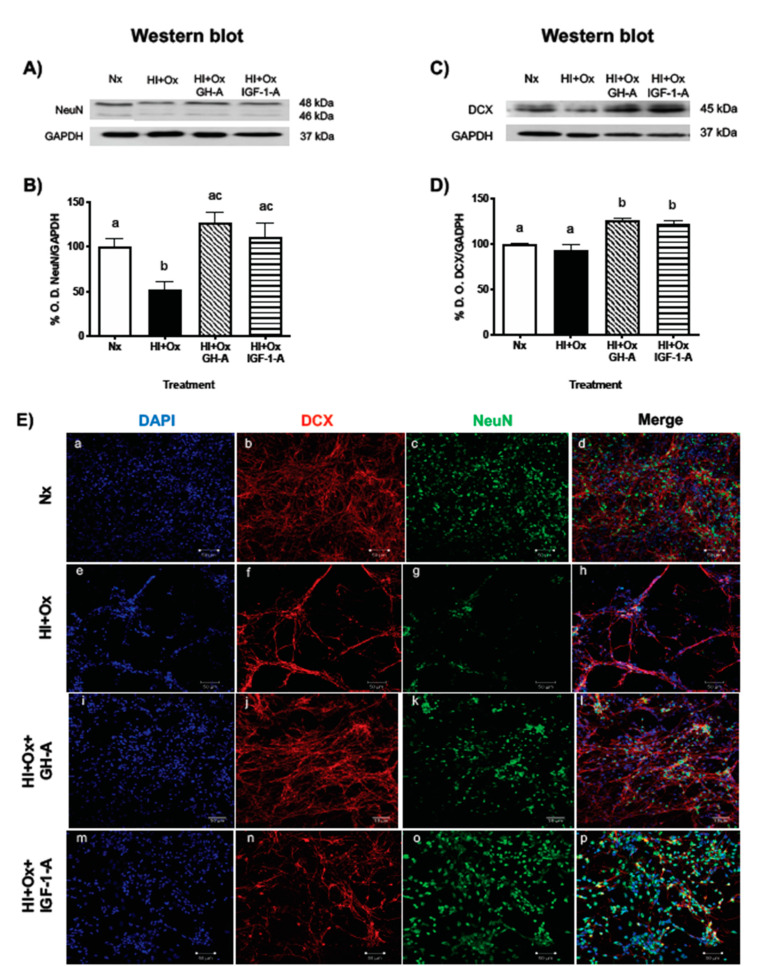
Treatments with GH or IGF-1 increase the survival of mature neurons and neural precursors after a hypoxic-ischemic injury in primary cerebellar cultures. Cells were maintained under either normoxic conditions (Nx), submitted to hypoxic-ischemic injury for 12 h (HI) and then reoxygenated for 24 h (HI + Ox), or treated with 1 nM GH or 40 nM IGF-1 after (denoted by A) 12 h of HI, along the 24 h of reoxygenation period (HI + Ox GH-A; HI + Ox IGF1-A). The upper panels show Western blots with specific immunoreactive bands corresponding to either neuronal nuclei protein (NeuN, panel (**A**)), a marker for mature neurons, or doublecortin (DCX, panel (**C**)), a marker for neuronal precursors, and GAPDH (panels (**A**,**C**)), used as loading control. Panels (**B**,**D**) show the densitometric (%O.D.) analysis of changes observed in panels (**A**,**C**). Both GH and IGF-1 treatments induced a significant increase of NeuN-IR (panel (**B**)) and DCX-IR (panel (**D**)). Bars represent the mean ± SEM, *n* = 4 independent experiments performed in duplicate. Groups with different letters are significantly different by one-way ANOVA and Tukey’s post hoc test (*p* < 0.01). (**E**) Representative confocal ICC images show the effects of different incubation conditions upon NeuN-IR, DCX-IR, and cell number (DAPI) in primary cerebellar cell cultures exposed to Nx (**Ea**–**d**), HI + Ox (**Ee**–**h**), HI + Ox plus GH treatment ((**Ei**–**l**) HI + Ox GH-A), and HI + Ox plus IGF-1 treatment (**Em**–**p**), HI + Ox IGF1-A). (**Ea**,**e**,**i**,**m**) shows cell nuclei counterstained with DAPI to determine the number of cells present in cultures; (**Eb**,**f**,**j**,**n**) shows DCX-IR; (**Ec**,**g**,**k**,**o**) shows NeuN-IR; and (**Ed**,**h**,**l**,**p**) shows the co-localization of NeuN-IR, DCX-IR, and DAPI in the merged images. Scale bar: 50 µm.

The protective effects of GH and IGF-1 upon neuronal subpopulations in primary cerebellar cell cultures exposed to hypoxic-ischemic conditions were analyzed by determining the expression of NeuN (mature neurons) and DCX (neuronal precursors) markers. As it is shown in Figure 4A,B, exposure of cultures to HI + Ox conditions resulted in a significant decrease (52.5 ± 8.7%, *p* < 0.01) in NeuN immunoreactive (IR) bands in relation to the Nx control (100 ± 9.1%), as analyzed by Western blot. However, the addition of either 1 nM rcGH or 40 nM rhIGF-1 at the reoxygenation period, after the acute injury event, significantly blocked such effect (HI + Ox GH-A, 126 ± 11.5%, *p* < 0.0004; HI + Ox IGF-1-A, 111.6 ± 15.1%, *p* < 0.01, respectively) and restored the expression of NeuN levels (Figure 4B). On the other hand, Figure 4C,D shows that HI + Ox conditions did not modify the expression of DCX-IR band (93.3 ± 6.3%) in comparison to the Nx control (100 ± 0.25%). However, the administration of rcGH or rhIGF-1 after injury resulted in a significant increase for DCX-IR (HI + Ox GH-A: 125.4 ± 2.5%, *p* < 0.0001; HI + Ox IGF-1-A: 121.4 ± 4.4, *p* < 0.0002, respectively) in comparison to both the Nx and injured controls, indicating a stimulation of this neuronal precursor marker under these conditions.

When analyzed by ICC and confocal microscopy (Figure 4E), an important reduction in total cell number (estimated by DAPI staining) was observed when cultures were exposed to HI + Ox conditions (Figure 4Ee) in comparison to the Nx controls (Figure 4Ea). Moreover, relevant decreases in DCX-IR (Figure 4Ef) and NeuN-IR (Figure 4Eg) were found in relation to their corresponding Nx controls (Figure 4Eb,c, respectively), and apparently the deleterious effect was more evident upon the mature neurons. As described before (Figure 2), disruption of cell interactions and clumping were also observed here (Figure 4Eh vs. Figure 4Ed). Confocal images show that treatment of the injured cells with either GH or IGF-1 prevented the damage provoked by hypoxia-ischemia and stimulated the survival of both DCX-IR and NeuN-IR neurons, as shown in Figure 4Ej,n,l and Figure 4Ek,o,p, respectively.

### 2.5. Effects of GH and IGF-1 Administration after Hypoxia-Ischemia upon PI3K/Akt, MAPK/ERK1/2, and Bcl-2 Pathways

**Figure 5:**
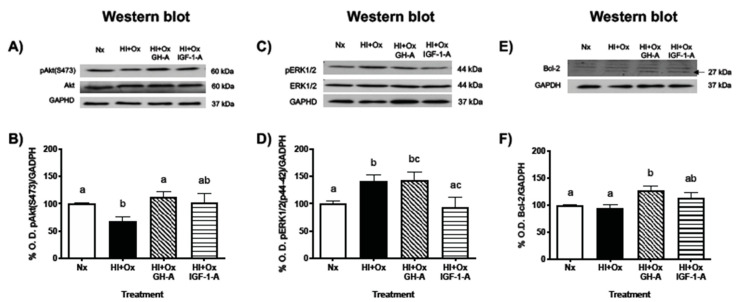
Effects of GH and IGF-1 treatments upon activation of the PI3K/Akt and MAPK/ERK1/2 signaling pathways and Bcl-2 expression after a hypoxic-ischemic injury in primary cerebellar cultures. Cells were maintained under either normoxic conditions (Nx), submitted to hypoxic-ischemic injury for 12 h (HI) and then reoxygenated for 24 h (HI + Ox), or treated with 1 nM GH or 40 nM IGF-1 after (denoted by A) 12h of HI, along the 24 h of reoxygenation period (HI + Ox GH-A; HI + Ox IGF-1-A). The upper panels show Western blots with specific immunoreactive bands corresponding to the following proteins: (**A**) total Akt (60 kDa) or pAkt (S473, 60 kDa); (**C**), ERK1/2 (44 kDa) or pERK1/2 (T202/Y204, 44 kDa); (**E**), Bcl-2 (27 kDa); and GAPDH (panels (**A**,**C**,**E**), 37 kDa) as loading control. (**B**,**D**,**F**) show densitometric (% O.D.) analysis of changes observed in (**A**,**C**,**E**). Bars represent the mean ± SEM, *n* = 5 independent experiments performed in duplicate. Groups with different letters are significantly different by one-way ANOVA and Tukey’s post hoc test (*p* < 0.01).

The effects of HI + Ox injury, as well as further treatment with GH or IGF-1, upon activation of several signaling pathways involved in cell survival, were evaluated by Western blotting, where we analyzed the phosphorylation ratio of Akt and ERK1/2, and the expression of Bcl-2 immunoreactivities. As it is shown in Figure 5A,B, the ratio of optical density (% O.D.) between pAkt(S473)/GAPDH significantly decreased in the HI + Ox group (67.6 ± 7.8%, *p* < 0.005) in comparison with the Nx group (100 ± 1.8%). On the other hand, the addition of GH resulted in a meaningful increase of Akt phosphorylation (HI + Ox GH-A, 113.2 ± 8.9%, *p* < 0.0002) in relation to the injured group, while the administration of IGF-1 restored pAkt to Nx levels, but this was not significantly different from the damaged control (HI + Ox IGF-1-A, 102.5% ± 16.8%). In contrast, Figure 5C,D shows that exposure of cerebellar cultures to HI + Ox conditions provoked a significant increase in the ratio of phosphorylated ERK1/2/GADPH (141.6 ± 10.8%, *p* < 0.03) in comparison to the Nx control (100 ± 5.5%), and GH treatment did not show any effect (HI + Ox GH-A, 143.4 ± 15.4%) in relation to the injured group but was significantly different (*p* < 0.02) from the normal controls. Instead, IGF-1 treatment significantly decreased the ratio of pERK1/2 (HI + Ox IGF-1-A, 93.6 ± 17.6%, *p* < 0.02) in relation to the HI + Ox group. Figure 5E,F shows that the antiapoptotic Bcl-2-IR band did not change in the HI + Ox group (94.6 ± 5.5%) in relation to the Nx control (99.1 ± 2.6%). However, Bcl-2-IR was significantly increased with GH treatment (HI + Ox GH-A, 127.7 ± 8.8%, *p* < 0.01) in comparison to both Nx and injured groups, whereas IGF-1 administration had no significant effects (HI + Ox IGF-1-A, 114.3 ± 9.7%) between groups.

### 2.6. Effects of Acute (HI) and Subacute (HI + Ox) Injury upon Local GH, GHR, IGF-1 and IGF-1R mRNAs Expression in Primary Cerebellar Cultures

**Figure 6:**
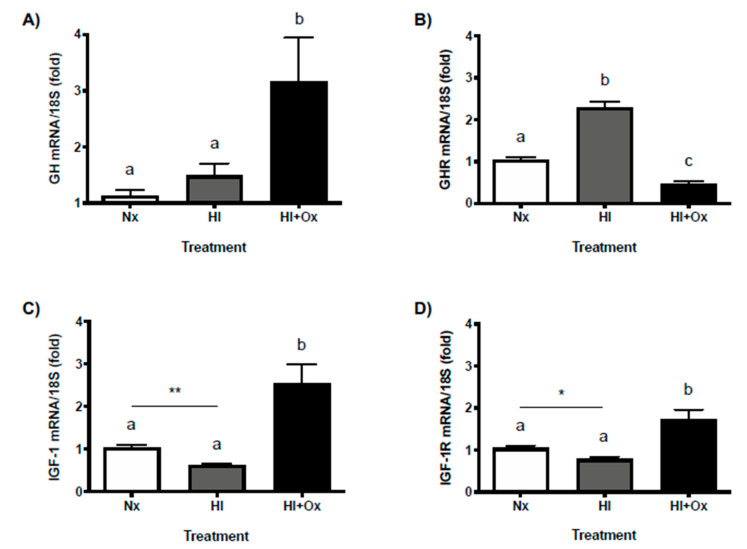
Expression of GH, GHR, IGF-1, and IGF-1R mRNAs in primary cerebellar cultures exposed to normoxic (Nx), acute hypoxia-ischemia (HI) injury, or subacute hypoxia-ischemia (HI + Ox) injury. The relative expression of (**A**) GH, (**B**) GHR, (**C**) IGF-1, and (**D**) IGF-1R mRNAs was determined by qPCR and corrected by the threshold cycle (CT) using the formula 2^-∆∆CT^. Ribosomal 18S RNA was used as the housekeeping gene control. Bars represent the mean ± SEM, *n* = 3 independent experiments by duplicate. Groups with different letters are significantly different by one-way ANOVA and Tukey’s post hoc test (*p* < 0.01). An unpaired Student’s *t* test was used to evaluate the effect of Nx versus HI conditions (panels (**C**,**D**)). Asterisks indicate significant differences between groups (* *p* < 0.05, ** *p* < 0.003).

Figure 6A shows that local GH mRNA expression was significantly increased mainly under the subacute injury condition (HI + Ox, 3.2 ± 0.7-fold, *p* < 0.006) as compared with Nx control (1.1 ± 0.1-fold), whereas as shown in Figure 6B, the GH receptor (GHR) mRNA expression was appreciably augmented during the acute injury phase (HI, 2.3 ± 0.1-fold, *p* < 0.0001), and then it significantly diminished after reoxygenation (HI + Ox, 0.47 ± 0.06-fold, *p* < 0.001), in relation with the Nx group (1.04 ± 0.05-fold). On the other hand, under HI conditions, the expression of local IGF-1 (0.62 ± 0.03-fold, *p* < 0.003, Figure 6C) and IGF-1R (0.78 ± 0.05-fold, *p* < 0.05, Figure 6D) mRNAs were substantially decreased, as compared to the Nx control (1.04 ± 0.06-fold and 1.04 ± 0.05-fold, unpaired t-test, respectively). Conversely, during the subacute phase injury phase (HI + Ox), the expression of both IGF-1 (2.5 ± 0.46-fold, *p* < 0.0005, Figure 6C) and IGF-1R (1.7 ± 0.2-fold, *p* < 0.003, Figure 6D) mRNAs significantly increased, in relation to the Nx controls.

### 2.7. Effects from GH and IGF-1 Gene Silencing by Specific siRNAs in Cerebellar Cell Cultures under Normal and Hypoxic-Ischemic Conditions, upon GH, GHR, IGF-1 and IGF-1R mRNAs Expression

To analyze the involvement of locally produced GH and IGF-1 in the protective response against HI injury, their respective mRNA expression was blocked using specific small-interfering RNAs (siRNAs). The mediated downregulation of both siGH and siIGF-1 in cerebellar cultures was determined by real-time PCR (qPCR) after 48 h post-transfection of the corresponding siRNAs.

**Figure 7:**
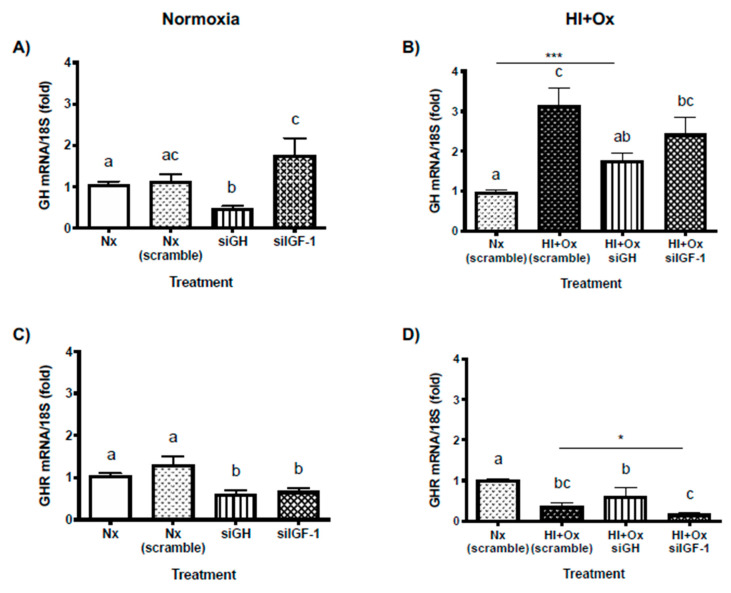

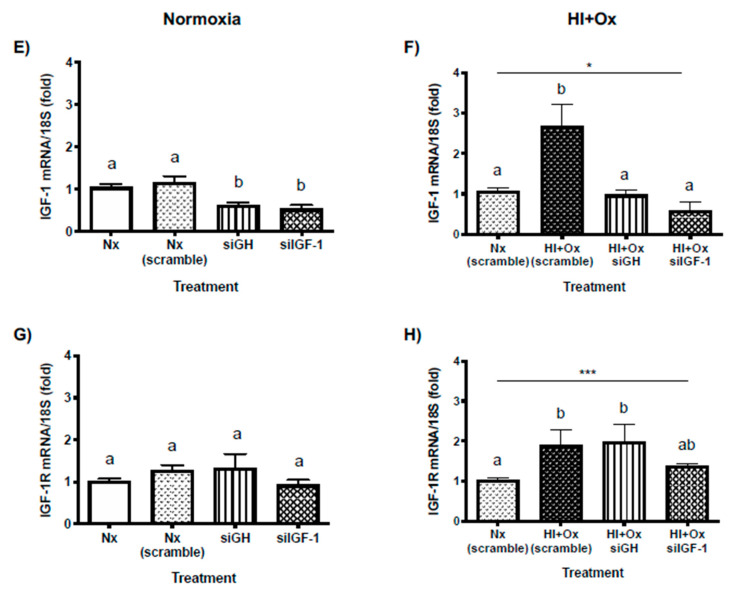
Effects of transfecting specific siRNAs (siGH or siIGF-1) to knock-down GH or IGF-1 gene expression in the primary cerebellar cultures exposed to either normoxic (Nx, panels (**A**,**C**,**E**,**G**)) or subacute hypoxic-ischemic injury (HI + Ox, panels (**B**,**D**,**F**,**H**)) conditions, upon local expression of GH (panels (**A**,**B**)), GHR (panels (**C**,**D**)), IGF-1 (panels (**E**,**F**)), and IGF-1R (panels (**G**,**H**)) mRNAs. Nonsilencing siRNA (scramble) was transfected to cells as an additional control. The relative expression was determined by qPCR and corrected by the threshold cycle (CT) using the formula 2^−∆∆CT^. Ribosomal 18S RNA was used as housekeeping gene control. Bars represent the mean ± SEM, *n* = 4 independent experiments by duplicate. Groups with different letters are significantly different by one-way ANOVA and Tukey’s post hoc test (*p* < 0.01). An unpaired Student’s *t* test was used to evaluate the effect of Nx scramble versus HI + Ox siGH conditions (panel (**B**)), HI + Ox scramble versus HI + Ox siIGF-1 (panel (**D**)), and Nx scramble versus HI + Ox siIGF-1 (panels (**F**,**H**)). Asterisks indicate significant differences between groups (* *p* <0.05, *** *p* <0.001).

Initially, the efficacy of mRNA knock-down was studied under Nx incubation conditions. A random oligonucleotide (Nx scramble) was used as an additional siRNA (negative) control. Results showed that, as expected, administration of siGH significantly reduced the expression of cerebellar GH mRNA (0.48 ± 0.04-fold, *p* < 0.004, Figure 7A), and also of IGF-1 mRNA (0.61 ± 0.07-fold, *p* < 0.009, Figure 7E), as compared with their corresponding Nx siRNA controls (1.13 ± 0.15-fold, and 1.16 ± 0.13-fold, respectively). Moreover, transfection with siGH induced a substantial decrease of GHR mRNA expression (0.61 ± 0.07-fold, *p* < 0.004, Figure 7C) in relation to its control group (1.2 ± 0.12-fold), but it had no effect upon IGF-1R mRNA expression (1.34 ± 0.32-fold, Figure 7G), which was no different from the Nx siRNA control (1.3 ± 0.18-fold). On the other hand, as anticipated, transfection with siIGF-1 significantly lowered the expression of IGF-1 mRNA (0.52 ± 0.08-fold, *p* < 0.0004, Figure 7E) in relation to the corresponding controls, but it induced a considerable increase of GH mRNA expression (1.76 ± 0.4-fold, *p* < 0.04, Figure 7A), compared to its Nx control (1.05 ± 0.07-fold), although it was not statistically different from the Nx siRNA group (1.13 ± 0.15-fold). Additionally, siIGF-1 provoked a clear reduction of GHR mRNA expression (0.67 ± 0.06-fold, *p* < 0.01, Figure 7C) relative to the controls, but it had no effect upon IGF-1R mRNA expression (0.93 ± 0.11-fold, Figure 7G).

Later, the effect of local GH and IGF-1 mRNAs knockdown was studied under HI + Ox conditions. In agreement with previous results (Figure 6A), the exposition of scramble-transfected cerebellar cells to hypoxic-ischemic injury resulted in a very strong and meaningful rise in the local expression of GH mRNA (HI + Ox scramble, 3.16 ± 0.41-fold, *p* < 0.0001, Figure 7B) as compared to the Nx scramble group (0.97 ± 0.04-fold). In addition, under these conditions, significant increases in the expression of IGF-1 (2.68 ± 0.51-fold, *p* < 0.0008, Figure 7F) and IGF-1R (1.89 ± 0.37-fold, *p* < 0.04, Figure 7H) mRNAs were found, in comparison to their corresponding Nx scramble controls. In contrast, HI + Ox scramble conditions provoked a substantial reduction of GHR mRNA expression (0.37 ± 0.07-fold, *p* < 0.0001, Figure 7D). Transfection of cerebellar cells with siGH provoked a significant diminution of GH mRNA expression (HI + Ox siGH, 1.77 ± 0.18-fold, *p* < 0.005, Figure 7B) with respect to the HI + Ox scramble group response, although it did not reach the levels obtained in the Nx scramble control (*p* > 0.001 unpaired *t* test). In addition, under these conditions, the expression of IGF-1 mRNA was appreciably decreased (0.98 ± 0.1-fold, *p* < 0.005, Figure 7F) in comparison to the HI + Ox group, and it returned to similar levels as in the Nx scramble control. The knockdown of GH by siGH maintained the GHR mRNA expression reduced (0.62 ± 0.2-fold, *p* < 0.01, Figure 7D) in relation to the Nx scramble control, and it was no different from the HI + Ox group, whereas the expression of IGF-1R was similar (1.97 ± 0.42-fold, *p* < 0.02, Figure 7H) to that observed in the hypoxic-ischemic control, and it remained significantly increased in comparison to the Nx scramble group. On the other hand, the transfection with siIGF-1 effectively provoked a substantial decrease of IGF-1 mRNA expression (0.59 ± 0.2-fold, *p* < 0.0002, Figure 7F) in comparison to the HI + Ox scramble group, and to levels lower than the Nx scramble control (*p* < 0.02, unpaired *t* test). The knockdown of IGF-1 did not affect the rise of GH mRNA expression produced by hypoxic-ischemic conditions, which was kept significantly augmented (2.43 ± 0.42-fold, *p* < 0.0008, Figure 7B) in comparison to the Nx scramble control. In addition, siIGF-1 provoked a meaningful reduction of GHR mRNA expression (0.17 ± 0.03-fold, *p* < 0.0001, Figure 7D) with respect to Nx scramble and HI + Ox scramble (*p* < 0.02, unpaired *t* test) conditions as well as to the HI + Ox siGH group. Finally, the expression of IGF-1R mRNA was different (1.37 ± 0.06-fold, Figure 7H) from Nx scramble (*p* < 0.009, unpaired *t* test) but not from HI + Ox scramble group.

### 2.8. Role of Locally-Expressed and Exogenously-Added GH and IGF-1 in the Neuroprotective Response to Hypoxic-Ischemic Injury in Primary Cerebellar Cultures

**Figure 8:**
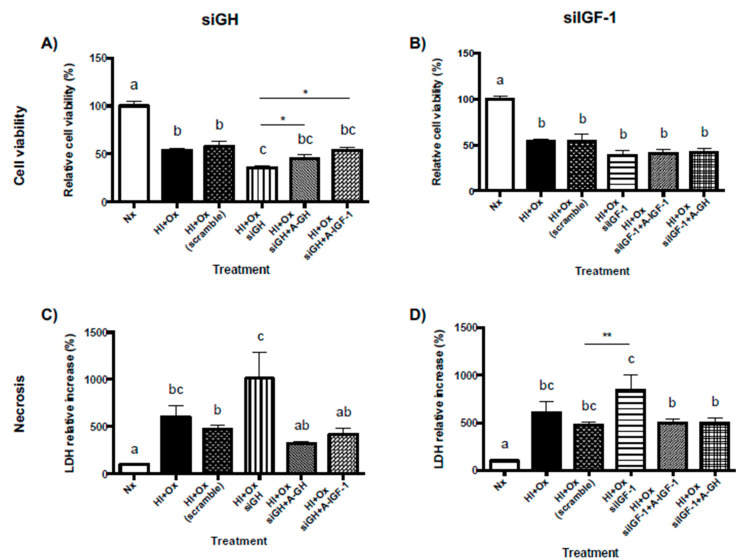
Neuroprotective actions of GH treatment applied after an acute hypoxic-ischemic injury (HI) upon cell viability and necrosis are partially mediated by IGF-1. Cerebellar cells that were transfected with either siGH (panels (**A**,**C**)) or siIGF-1 (panels (**B**,**D**)) were treated with 1 nM GH or 40 nM IGF-1 in a subacute hypoxic-ischemic injury (HI + Ox). Nonsilencing siRNA (scramble) was transfected to cells as an additional control. Cell viability was evaluated by the MTT assay (panels (**A**,**B**)), whereas cell necrosis was determined by LDH activity released to the culture media (panels (**C**,**D**)). Bars represent the mean ± SEM, *n* = 4 independent experiments by duplicate. Groups with different letters are significantly different by one-way ANOVA and Tukey’s post hoc test (*p* < 0.01). An unpaired Student’s *t* test was used to compare HI + Ox siGH versus HI + Ox siGH GH-A and HI + Ox siGH IGF-1-A groups (panel (**A**)) and HI + Ox scramble versus HI + Ox siIGF-1 groups (panels (**B**,**D**)). Asterisks indicate significant differences (* *p* <0.05, ** *p* <0.006).

Figure 8A shows that both HI + Ox and HI + Ox scramble groups provoked a substantial decrease of cell viability (54.08 ± 2.1%, *p <* 0.0001, and 57.6 ± 5.7%, *p <* 0.0001, respectively) as compared to the Nx control (100 ± 4.5%). Moreover, knocking-down GH mRNA expression provoked a further significant reduction in cell viability (35.8 ± 2.1%) in comparison with the HI + Ox scramble group (*p* < 0.01) and with the Nx control (*p* < 0.0001). However, treatment with exogenous rcGH (1 nM) partially restored and appreciably increased cell viability (HI + Ox siGH + GH-A, 45.3 ± 3.8%, *p* < 0.02, unpaired *t* test). Likewise, the addition of external IGF-1 (40 nM) promoted a partial rescue and significant increase of cell viability (HI + Ox siGH + IGF-1-A, 53.7 ± 3.2%, *p* < 0.0001) in comparison with the HI + Ox siGH group. Figure 8C shows that the exposure of cells to HI + Ox and HI + Ox scramble conditions induced a strong and significant necrotic response (606 ± 116.7%, *p* < 0.0007, and 474.0 ± 37.3%, *p* < 0.05, respectively), in comparison to the Nx control (100 ± 5.4%), as determined by LDH release to the culture media. Interestingly, knocking-down local GH expression further increased the necrotic response almost twice (1,011 ± 276.6%, *p* < 0.02) in relation to that observed in the HI + Ox scramble group. Again, the treatment with exogenous GH or IGF-1 significantly decreased LDH release and necrosis (320.1 ± 25.4%, *p* < 0.02, and 421.7 ± 62.2%, *p* < 0.04, respectively), indicating a role of these factors in rescuing cells from necrotic death.

On the other hand, as shown in Figure 8B, neither the treatment with exogenous IGF-1 (HI + Ox siIGF-1 + IGF1-A, 40.9 ± 4.4%) or GH (HI + Ox siIGF-1 + GH-A, 41.8 ± 4.1%) significantly modified the deleterious effect of the knockdown of local IGF-1 expression (HI + Ox siIGF-1, 38.9 ± 5.1%) in comparison with the HI + Ox scramble upon cell viability (54.1 ± 2.1%, *p* < 0.05, unpaired *t* test). However, as shown in Figure 8D, knocking-down local IGF-1 mRNA expression showed a substantial increase of necrosis (HI + Ox siIGF-1, 842.9 ± 161.7%, *p* < 0.006, unpaired *t* test) in relation to the HI + Ox scramble group (474 ± 37.3%) and to Nx conditions (100 ± 5.4%, *p* < 0.0001). Treatment with either IGF-1 (HI + Ox siGH + IGF-1-A, 498.2 ± 39.6%, *p* < 0.041) or GH (HI + Ox siGH + GH-A, 494.6 ± 57.1%, *p* < 0.02) significantly reduced LDH release in comparison to the HI + Ox siIGF-1 group, indicating that these factors partially rescued the cells from necrosis.

## 3. Discussion

Here, we show that the primary neuronal cultures from cerebellum (neuronal precursors and mature neurons) that were exposed to hypoxia-low glucose or oxygenation damage improve their viability and survival parameters when they are incubated in the presence of GH and IGF-1. In addition, our findings support that the endogenous expression of GH and IGF-1 during an acute hypoxic-ischemic injury is essential to maintaining cell survival. Moreover, we demonstrated that the GH-dependent IGF-1 mRNA expression during an acute injury partially mediates the protective effects of GH in primary cerebellar neurons. Taken together, these results provide further evidence for the autocrine/paracrine mechanisms of GH and IGF-1 that mediate their neuroprotective effects.

Our findings that the expression of the transcription factor hypoxia-inducible factor 1α (HIF-1α) increases after a hypoxia-ischemia insult supports that cerebellar primary neurons are responsive to the injury conditions and are consistent with previous studies, both in vivo and in vitro, showing that mouse cortical neurons exposed to hypoxic conditions showed an increase in this factor [32,33,34]. It is well known that the increase of HIF-1α is a characteristic response of cells to hypoxia and ischemia during early development [35,36], which could be associated with its neuroprotective effects [37], although it has also been described that the increase has neurotoxic effects [38,39]. Our results show that cell viability decreased after exposure to hypoxia-ischemia and reoxygenation while cell death parameters (apoptosis and necrosis) increased, which suggests that the intrinsic factors of primary cerebellar neurons are not sufficient to decrease the cell damage progression.

We previously found that GH treatment improves the viability and survival parameters of cerebellar primary neurons when it is added during the hypoxia-ischemia insult [11]. In order to know whether GH is also capable of conferring neuroprotection during the damage produced by reoxygenation and to investigate the role that IGF-1 plays in this process, in the present work, we analyzed the phase-dependent impact of GH and IGF-1 on neuronal cell death. Our results show that both GH and IGF-1 improve viability and inhibit cell death in both stages of cell injury, which agree with previous studies reporting that GH and IGF-1 have important roles as neurotrophic factors [3,11,18,40,41,42].

It was described that the severity of damage caused by hypoxia depends on the state of neuronal differentiation [43]. To extend our understanding of the mechanisms behind the positive effects of GH and IGF-1 on cell survival, we also evaluated the impact of GH and IGF-1 on the mature and immature neuronal populations. We observed a significant reduction in the mature neural cells (NeuN positive) compared with the immature population (DCX positive) under HI + Ox conditions, which is consistent with our previous observation [11]. Specifically, the susceptibility of the NeuN positive cells to death mainly by necrosis, along with apoptosis, due to HI + Ox, is related to the depletion in cellular ATP levels [18,43]. Interestingly, we observed a significant increment in the NeuN positive and DCX positive cells from the primary cerebellar cell cultures treated with GH and IGF-1 during the oxygenation phase. These observations are consistent with previous reports describing that GH has protective actions in both mature neurons [44,45] and in primary neurospheres derived from embryonic neural stem cells of mouse [46,47]. Sanchez-Bezanilla et al. [48] observed that GH promotes cell proliferation (BrdU-positive cells) within the peri-infarct regions in stroke mice. Furthermore, they tracked the fate of these proliferating cells and found an increase in the number of NeuN and DCX positive cells. These findings further support that GH plays an important role in neurogenesis in a stroke model. Similarly, Lin et al. [49] found an IGF-1-dependent increase in the number of DCX and NeuN positive cells and concluded that in addition to its antiapoptotic effects, IGF-1 acts as a mitogenic agent in a neonatal rat hypoxic-ischemic model of brain injury, which is in accordance with the amount of evidence supporting the claim that IGF-1 plays a key role in the proliferation and maturation of neurons, both in vivo and in vitro [50,51].

The mechanisms by which GH exerts its protective effects include the suppression of apoptotic related proteins (Bax) or increasing the expression of antiapoptotic proteins such as Bcl2 and activation of the PI3K/AKT signaling pathway [11,41,52]. Our analysis confirmed that AKT phosphorylation, which decreased in hypoxic condition, is recovered by GH treatment, while Bcl2 expression increases in response to GH. In addition, we found that although these were not statistically significant, the absolute levels of phosphorylated AKT and Bcl2 expression increased with the IGF-1 treatment, which is consistent with the numerous evidence, indicating that IGF-1 is an activator of the PIK3/AKT signaling pathway as a mechanism to promote cell survival and inhibit apoptosis [14,20,53,54,55,56,57].

The activation of the ERK1/2 signaling pathway is recognized as a response of neurons displaying signs of damage after a hypoxic-ischemic insult [58,59,60] and our results confirmed that ERK1/2 phosphorylation is increased in cerebellar primary neurons during reoxygenation. However, we observed that ERK phosphorylation does not increase with GH treatment beyond the point that it increases with the oxygenation damage. This may indicate that the activity of the ERK1/2 signaling pathway observed during the cell damage is the maximum response, and this cannot be enhanced by GH treatment. Conversely, although previous studies demonstrated that IGF-1 is a strong activator of the ERK1/2 pathway [56,57], we found that IGF-1 treatment blocked ERK1/2 phosphorylation induced by neuronal damage. This suggests that IGF-1 may exert its effects at other time points not assessed or that treatment saturates the ERK1/2 response.

We analyzed changes in the expression of GH and IGF-1 mRNAs (and their respective receptors) in order to study the role of the endogenous GH-IGF-1 system during hypoxia and oxygenation damage. Our results showed that this hormonal system increases its local expression in response to cell damage, mainly during reoxygenation, although it is important to note that GHR expression decreases in this condition, perhaps as a negative feedback mechanism. These results are consistent with the numerous evidence that indicates that the endogenous expression of GH and IGF-1 during a damaging condition, whether due to hypoxia [11,61], excitotoxicity [12,62], or trauma [13,63], plays a critical role within the mechanisms triggered by neurons to preserve their survival and viability.

The binding of GH to its specific receptor causes an increase in the synthesis of IGF-1, which is released and, in turn, binds to its specific receptor. Thus, GH has direct effects and other effects mediated by IGF-1 [20,64,65]. By silencing GH and IGF-1 mRNAs, we tested how the GH-IGF-1 axis was affected by GH or IGF-1 deficiency and how this impacted the survival parameters of primary cerebellar neurons exposed to hypoxic and oxygenation injury. We confirmed that GH silencing leads to the downregulation of GHR and IGF-1 mRNAs, while IGF-1 deficiency impacts negatively on the GHR mRNA levels. These findings are consistent with previous reports [66,67,68] which demonstrated that GH deficiency in parallel affects the expression of its receptor. On the other hand, we discovered that the effect of oxygenation damage on the increased expression of IGF-1 is completely blocked by GH silencing, while the silencing of IGF-1 had no impact on the hypoxia-dependent GH induced expression. These findings demonstrated that the protective response of the endogenous GH-IGF-1 system depends, initially, on the expression of GH, which support the hypothesis that, in addition to the endocrine contribution of GH, the autocrine–paracrine mechanisms of GH also play an important role during the neuroprotective response of cerebellar neurons.

Our findings on cell viability and necrosis show that GH deficiency exacerbates the damage produced by oxygenation (seen as relative cell viability and LDH release), which is blocked by GH or IGF-1 exogenous treatment. Previous works by our group [11,17] and others [63,69] have pointed out the importance of GH as a neurotrophic factor, which have direct effects and other effects mediated by IGF-1 and other neurotrophins, including BDNF, NT3, or VEGF [7,11,63,69]. This explains our results, which show that IGF-1-deficient neurons treated with GH improve their viability, likely through the regulation of other neurotrophins. Intriguingly, GH or IGF-1 replacement treatment in IGF-1 silencing neurons also rescued neurons from oxygenation damage (seen as LDH release), which is further evidence that IGF-1 is important during GH-mediated neuroprotection, but that GH can also trigger other mechanisms to confer neuroprotection.

Taken together, our findings confirm that cerebellar neurons in damaged conditions trigger protection mechanisms that include an increase in GH and IGF-1 expression, which, through autocrine–paracrine mechanisms, contributes to preserving cell integrity and viability. Furthermore, treatment with exogenous GH represents a substantial improvement in the recovery of cell viability parameters, through mechanisms including the activation of survival signaling pathways, the positive regulation of IGF-1, and likely, the up-regulation of other neurotrophins such as BDNF, NT3, or VEGF. GH therapy is a promising opportunity to treat neural injuries and diseases; however, long-term side effects of GH treatments are still largely unknown and cannot be ruled out. Thus, this novel field of research deserves further work.

## 4. Materials and Methods

### 4.1. Animals

Pathogen-free, fertilized eggs (*Gallus gallus*, White Leghorn) were obtained from Pilgrim’s Pride (Querétaro, México) and were incubated at 39 °C in a humidified air chamber (IAMEX, México). The eggs were rotated one-quarter of a revolution every 50 min during incubation. The chicken embryos were sacrificed by decapitation according to a protocol approved by the bioethical committee of the Instituto de Neurobiología, UNAM, and in accordance with the Mexican official regulation (NOM-062-ZOO-1999, 10/19/2010).

### 4.2. Primary Cerebellar Cell Culture

Chicken embryos at 15 days of embryogenesis (15 DE) were anesthetized in ovo by cooling them on ice for 5 min and then sacrificed by decapitation. Cerebellums were collected and put into a cold calcium/magnesium-free Hank’s balanced buffer. Then, they were treated with 0.002% trypsin in DMEM (Sigma-Aldrich, St. Louis, MO, USA), at 39 °C for 20 min, followed by mechanical dissociation with a siliconized Pasteur pipette of narrow bore size and passed through a 40 µm nylon mesh. Later, 7.5 × 10^5^ cerebellar cells were plated on 50 µg/mL poly-l-lysine-coated 12-well culture plates and grown in a Neurobasal medium (containing 4.5 g/L glucose) added with 2% B27 supplement, 0.5 mM l-glutamine, and 1% antibiotic-antimycotic mix (amphotericin B, penicillin, streptomycin; all from Gibco-BRL, Burlington, ON, Canada) at 39 °C in a humidified atmosphere of 95% air and 5% CO_2_ chamber for 48 h prior to treatments [11].

### 4.3. Treatments

Experimental hypoxic-ischemic (HI) conditions were induced by replacing the Neurobasal-B27 culture medium with DMEM-low-glucose (LG) 1x media (1 g/L, Gibco-BRL), and the cultures were then placed in a humidified hypoxic chamber (Napco E Series, Model 302, Tualatin, OR, USA), at 37 °C, which was previously flushed with a mixture of 5% CO_2_ and 95% N_2_ for 10 min, resulting in a level of <5% O_2_ that was maintained for 12 h throughout the experiment, and continuously monitored with an ambient oxygen sensor (BW Technologies, Arlington, TX, USA). HI conditions were terminated by replacing the DMEM-LG medium with Neurobasal-B27 medium, and cultures were then incubated for an additional 24 h period of oxygenation (HI + Ox) under normoxic conditions (Nx, 95% air and 5% CO_2_).

In order to determine whether GH or IGF-1 were able to protect cerebellar cells from HI injury, the cultures were treated with either 1 nM recombinant chicken growth hormone (rcGH; Revholt, PRL, Israel), or a 40 nM recombinant human insulin-like growth factor-1 (rhIGF-1; BioVision, 4119-B9059, Malpitas, CA, USA) following the protocols depicted in Figure 1. Hormones were administered to the cell cultures in a Neurobasal medium with 0.02% B27, either during the 12 h acute injury (HI) incubation conditions (GH-D or IGF-1-D) or after, in the reoxygenation (sub-acute injury phase) period (HI + Ox GH-A; HI + Ox IGF-1-A) for another 24 h. The analyses were performed after this final time interval.

### 4.4. Determination of Cell Viability

After stabilization under normal conditions for 48 h at 39 °C, the primary cerebellar cell cultures were exposed to the different treatments (Nx, HI, HI + Ox, with or without the hormones). At the end, cell survival was determined by either of two methods:

(a) Trypan blue exclusion assay: Cells were harvested and resuspended in 1 mL medium, then a 10 µL aliquot was mixed with a 10 µL 0.05% trypan blue solution (Gibco 15250061, Grand Island, NY, USA), placed into a Neubauer chamber, and several fields were observed under a microscope (Olympus CX41). At least 100 cells (in duplicate) were analyzed for viability, and the mean percentage of living cells was calculated [70].
(b) MTT assay was performed according to manufacturer’s instructions: In brief, culture media in the plates were substituted with DMEM media without phenol red, and then 500 µL of MTT (3-[4,5-dimethylthiazol-2-yl]-2,5-diphenyltetrazolium bromide, Thermo-Fisher Scientific M6494, Waltham, MA, USA) labeling reagent, at a final concentration of 0.5 mg/mL, was added to each well and incubated for 4 h at 39 °C. The resulting formazan crystals were dissolved using an equal volume of the solubilization solution (1 g/mL SDS in 0.01 N HCl), and the plates were incubated for another 4 h in a humidified atmosphere at 39 °C. Aliquots (200 µL) of soluble formazan product were placed in a 96-well plate and optical density was analyzed at 570 nm in a microplate reader (Bio-Rad, Mod. 550, Hercules, CA, USA).

### 4.5. Determination of Apoptosis by Caspase-3 Activity

Apoptotic cell death in primary cerebellar cell cultures was analyzed by using a caspase-3 colorimetric assay kit (Assay Designs Inc., Ann Arbor, MI, USA). The samples (12 µg) of cell lysates from each treatment, i.e., standards, p-nitroaniline (pNA) standard, and blank controls, were placed in a 96-well plate. After 3 h of incubation at 37 °C, the reaction was stopped with 1 N HCl and then read at 405 nm in a microplate reader (Bio-Rad, Mod. 550, Hercules, CA, USA). The caspase-3 enzymatic activity was calculated as units per microgram of protein [71], normalized and expressed as the percentage of activity relative to the normoxic (Nx) controls.

### 4.6. Determination of Necrosis by Lactate Dehydrogenase (LDH) Release

LDH activity was determined following the Vassault method [72]. Briefly, at the end of treatments, 200 µL of culture media were mixed with 2.3 mL of Tris/NaCl/NADH buffer (81.3 mM Tris, 203.3 mM NaCl, 0.244 mM NADH, pH 7.2), and 500 µL of Tris/NaCl/pyruvate (81.3 mM Tris, 203.3 mM NaCl, 9.76 mM pyruvate, pH 7.2) solution; then, the decrease of absorbance at 340 nm, due to the oxidation of NADH, was recorded every minute for 5 min in a Beckman Coulter-DU 720 UV/Vis scanning a spectrophotometer (Brea, CA, USA). The enzymatic activity was calculated as the relative activity (Units/mL, µmoles/min/mL), normalized and expressed as percent of the Nx controls.

### 4.7. Immunocytochemistry

For the immunocytochemical analysis (ICC), 5 × 10^5^ cells were plated on a 10-mm culture fluorodish (World Precision Instruments Inc., Sarasota, FL, USA); after treatments, they were fixed with 4% paraformaldehyde in PBS, pH 7.4, for 30 min. The plates were then washed with PBS and permeabilized with 0.05% Triton X-100 in PBS. Double staining with specific antibodies (Table 1) and a confocal analysis were performed to study the effects of GH or IGF-1 treatments upon several neuronal cell sub-populations. The occurrence of doublecortin immunoreactivity (DCX-IR) was employed to identify neural precursors, while that of neuronal nuclear protein immunoreactivity (NeuN-IR) for mature neurons. Anti-DCX and anti-NeuN primary antibodies (Table 1) were diluted (1:1000) in TPBS with 1% nonfat dry milk (Bio-Rad, Hercules, CA, USA), then added to the plates and incubated overnight at room temperature (RT). After washing (3 × 15 min) in TPBS, plates were then incubated for 2 h, at RT with the corresponding secondary antibodies: goat anti-rabbit IgG-Cy3 antibody or rabbit anti-mouse IgG-FITC antibody (at dilutions mentioned in Table 1) in TPBS with 1% nonfat dry milk (Bio-Rad). Then, plates were washed (3 × 15 min) in TPBS and counterstained with 300 nM 4′6-diamidino-2-phenylindole (DAPI, Invitrogen) in PBS for 30 min, rinsed three times and stored in TBS. The plates were analyzed using a Carl Zeiss LSM 510 confocal microscope with lasers at excitation wavelengths of 561 nm (Cy3), and 488 nm (FITC), respectively. A coherent-XR multiphotonic laser at 350 nm was also employed for DAPI staining to determine total cell number.

In an independent set of experiments, the impact of HI conditions upon cerebellar cells was determined by analyzing the expression of hypoxia-inducible factor 1-alpha immunoreactivity (HIF-1α-IR), as well as the neuronal marker ß-Tubulin III, employing the specific antibodies listed in Table 1. The extent of the cell response to hypoxia-ischemia injury was measured as the ratio of HIF-1α-IR/DAPI relative area (%) in the plates.

### 4.8. Western Blot Analysis

Total proteins in primary cerebellar cell cultures were extracted using a homogenization buffer (50 mM HCl-Tris, pH 9.0), complemented with a protease inhibitor cocktail (Mini-complete, Roche, Basel, Switzerland). Equal amounts of protein (50 µg) were separated by SDS-PAGE in 12.5% slabs and transferred onto nitrocellulose membranes (Bio-Rad), as previously described [11]. Nitrocellulose-free binding sites were blocked with 5% nonfat dry milk (Bio-Rad) in TBS (Gibco, Grand Island, NY, USA) for 1 h, at RT. Membranes were then incubated overnight at 4 °C with the corresponding specific primary antibodies (Table 1), diluted in TTBS (0.05% Tween [*v*/*v*] in 1× TBS). After washing the membranes with TTBS (3 × 10 min), they were incubated for 2 h at RT with the corresponding HRP-conjugated secondary antibodies (Table 1). Chemiluminescent immunoreactive bands were developed using the ECL blotting detection reagent on autoradiography film (both from Amersham-Pharmacia, Buckinghamshire, UK). Kaleidoscope molecular weight markers (Bio-Rad, Cat. 1610375) were used as a reference for the molecular mass determination. In order to study the difference between the proportion of phosphorylated vs nonphosphorylated proteins among treatments in the same blots, a stripping method was used [11,71]. The DCX, NeuN, p-Akt, p-ERK1/2, and Bcl-2 immunoreactive bands intensities were normalized to GAPDH band intensity as the protein-loading control. Immunoblots were repeated up to 3 times, scanned, and quantified by densitometric analysis using ImageJ software (developed by NIH, freeware, [73]).

### 4.9. RT-PCR

Total RNA was extracted from each well (3 wells per experimental condition) by adding 1 mL of Trizol (Invitrogen, Waltham, MA, USA) according to the manufacturer’s directions. RNA was purified from cellular lysates using the Zymo Direct-zol purification kit according to instructions (Zymo Research Corp., Irvine, CA, USA). Genomic DNA contamination was removed by DNase I treatment (Invitrogen, Waltham, MA, USA) for 15 min at RT. The cDNA was synthesized from 1 µg of total RNA using 100 U of Superscript III Reverse Transcriptase (Life Technologies, Invitrogen, Waltham, MA, USA), 1 mM dNTPs, 0.5 µg oligo d(T), and 0.5 µg random hexamers, for 60 min at 42 °C.

### 4.10. Quantitative PCR (qPCR)

The expression of GH, GH receptor (GHR), IGF-1, and IGF-1 receptor (IGF-1R) mRNAs was quantified by quantitative PCR (qPCR) in a StepOne Real-Time PCR system (Applied Biosystems, Foster, CA, USA), and using SYBR Green (Roche, Mannheim, Germany) in a 10 µL final volume containing 3 µL cDNA (diluted 1:3 for GH, GHR, IGF-1, IGF-1R, and 1:5 for 18S), and 1 µL of each specific primer (0.5 µM). Primer sets used (Table 2) were designed to amplify avian mRNAs and to cross intro-exon boundaries to control for genomic DNA contamination. Reactions were performed under the following conditions: initial denaturation at 95 °C for 10 min, followed by 40 cycles at 95 °C for 15 s, 60 °C for 15 s, and 72 °C for 20 s. Dissociation curves were included after each qPCR experiment to ensure primer specificity. The relative abundance of GH, GHR, IGF-1, and IGF-1R mRNAs was calculated using the comparative threshold cycle (Ct) method and employing the formula 2^−∆∆CT^ [74], where the quantification is expressed relative to the geometric mean of 18S ribosomal mRNA [75].

### 4.11. Knockdown of GH and IGF-1 RNA Expression by Small-Interfering RNA (siRNA)

GH siRNA (5′-(UUUAGUUUCUCAAACACUCUGUC)-3′), IGF-1 siRNA (5′-(UGAAGUAGAAGCCUCUGUCUCCACA)-3′), and scramble siRNA (5′-(UUUGGAGUAUCUCUACGGACCGAGG)-3′) were custom made (Thermo-Fisher Scientific, Waltham, MA, USA). The transfection protocol employed was previously characterized [76,77] with minor modifications. In brief, siRNA transfections were carried out in a 100 µL final volume per dish containing either 70 nM siRNA for GH, or 170 nM for IGF-1, with 2 µL of Lipofectamine RNAi MAX (Thermo-Fisher Scientific, Waltham, MA, USA). Lipofectamine and siRNAs were diluted independently in 48 µL of Opti-MEM media (Thermo-Fisher Scientific, Waltham, MA, USA) and incubated at RT for 5 min. To produce liposomes, siRNAs and lipofectamine dilutions were mixed during 20 min; then, the cell cultures were exposed to liposomes containing GH or IGF-1 siRNAs, and incubated for 48 h at 39 °C in a humidified chamber. Scramble siRNA was used as siRNA control.

### 4.12. Statistical Analysis

In all experiments, values are expressed as mean ± SEM. Significant differences between multiple groups were determined by one-way ANOVA and Tukey’s post hoc test. An unpaired Student’s *t* test was used to compare between two groups where appropriate; *p* values less than 0.05 were determined to be statistically significant (* *p* < 0.05; ** *p* < 0.01; *** *p* < 0.001).

## Figures and Tables

**Table 1:** Antibodies.

Target	Host/Type	Dilution	Source	Cat. No.
DCX	guinea pig/polyclonal	1:1000	Sigma-Aldrich	AB2253
NeuN	mouse/monoclonal	1:1000	Sigma-Aldrich	MAB377
HIF-1α	rabbit/polyclonal	1:1000	Cell Signaling	3716S
β-Tubulin III	mouse/monoclonal	1:2000	Sigma-Aldrich	T8578
Phospho-Akt(S437)	rabbit/monoclonal	1:1000	Thermo Fisher Scientific	44-621G
Phospho-p44/42 MAPK (Erk1/2) (Thr202/Tyr204)	rabbit/monoclonal	1:1000	Cell Signaling	4370S
Bcl-2	rabbit/polyclonal	1:1000	Invitrogen	138800
GAPDH	rabbit/monoclonal	1:2000	Cell Signaling Technology	14C10
Goat anti-mouse IgG (H + L) cross-adsorbed secondary antibody, HRP	goat/polyclonal	1:5000	Thermo Fisher Scientific	G-21040
Goat anti-rabbit Ig (H + L) secondary antibody, HRP	goat/polyclonal	1:5000	Invitrogen	656120
Goat anti-guinea pig IgG antibody, HRP conjugate	goat/polyclonal	1:5000	Millipore	AP108P
Rabbit anti-mouse IgG (H + L) secondary antibody, FITC	rabbit/polyclonal	1:2000	Invitrogen	A16161
Goat anti-rabbit IgG antibody, Cy3 conjugate	goat/polyclonal	1:5000	Millipore	AP132C

**Table 2:** Oligonucleotides.

Target	Primer	Sequence (5′–3′)	Size	Accession Number
cGH	Fwd Rev	CGCACCTATATTCCGGAGGAC GGCAGCTCCATGTCTGACT	128 bp	NM_204359
cGHR	Fwd Rev	ACTTCACCATGGACAATGCCTA GGGGTTTCTGCCATTGAAGCTC	180 bp	NM_001001293.1
cIGF-1	Fwd Rev	TACCTTGGCCTGTGTTTGCT CCCTTGTGGTGTAAGCGTCT	170 bp	NM_001004384
cIGF-1R	Fwd Rev	TCCAACACAACACTGAAGAATC ACCATATTCCAGCTATTGGAGC	166 bp	NM_205032.1
c18S	Fwd Rev	CTCTTTCTCGATTCCGTGGGT TTAGCATGCCAGAGTCTCGT	100 bp	M59389

## Data Availability

The data that support the findings of this study are available from the corresponding author upon reasonable request.

## Supplementary Materials

The following are available online at https://www.mdpi.com/1422-0067/22/1/256/s1.
