# Supplementary material for: Neuroprotective Effects of Growth Hormone (GH) and Insulin-Like Growth Factor Type 1 (IGF-1) after Hypoxic-Ischemic Injury in Chicken Cerebellar Cell Cultures
Source: Int J Mol Sci. 2020 Dec 29;22(1):256. doi: 10.3390/ijms22010256 (PMC7795313; doi:10.3390/ijms22010256)
Supplement: Supplementary file 1 [file ijms-22-00256-s001.pdf]

International Journal of
Molecular Sciences

Supplementary Material

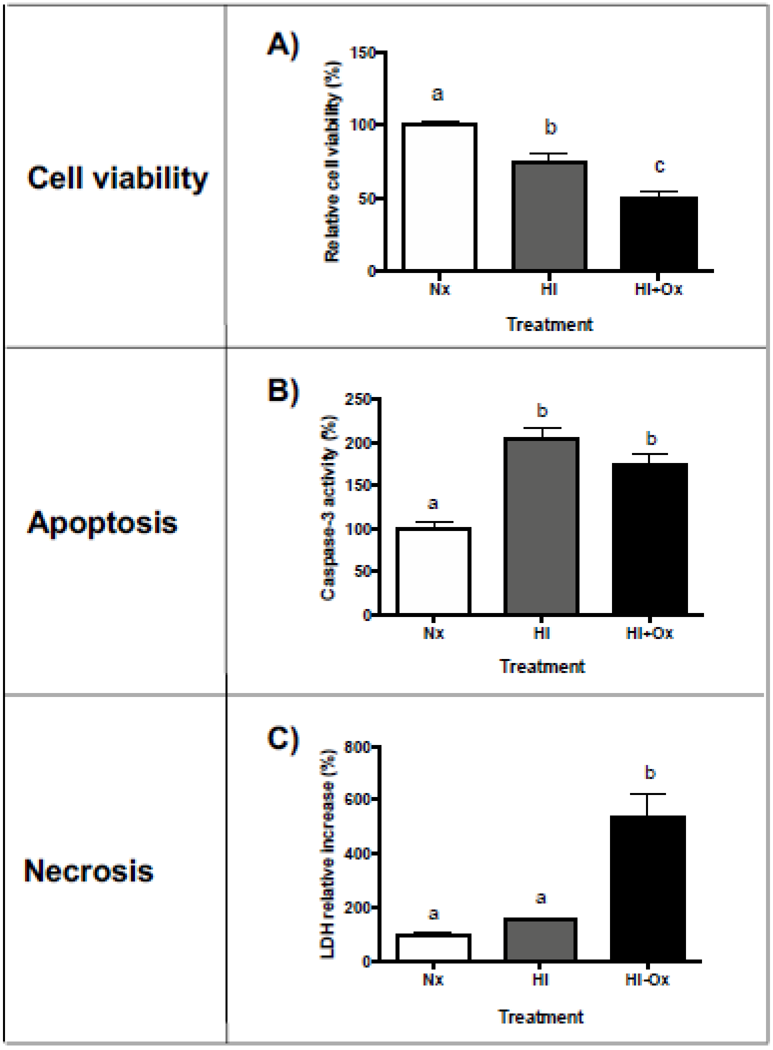

Figure S1. Determination of cell viability, apoptosis and necrosis in primary cerebellar cultures exposed to normoxic (Nx), acute hypoxia-ischemia (HI) injury, or sub-acute hypoxia-ischemia (HI+Ox) injury. A) Cell viability was analyzed by the trypan blue exclusion method. Bars represent the mean ± SEM, n=3 independent experiments performed by duplicate. B) Apoptosis was studied by measuring caspase-3 activity. Bars represent the mean ± SEM, n=5 independent experiments performed by duplicate. C) Necrosis was evaluated by analyzing the activity of lactate dehydrogenase (LDH) released to the culture media. Bars represent the mean ± SEM, n=5 independent experiments performed in duplicate. Groups with different letters are significantly different by one-way ANOVA and Tukey's *post-hoc* test ($p < 0.01$).
